# Kaiso-induced intestinal inflammation is preceded by diminished E-cadherin expression and intestinal integrity

**DOI:** 10.1371/journal.pone.0217220

**Published:** 2019-06-14

**Authors:** Shaiya C. Robinson, Roopali Chaudhary, Rodrigo Jiménez-Saiz, Lyndsay G. A. Rayner, Luke Bayer, Manel Jordana, Juliet M. Daniel

**Affiliations:** 1 Department of Biology, McMaster University, Hamilton, Ontario, Canada; 2 Department of Pathology & Molecular Medicine, McMaster Immunology Research Centre (MIRC), McMaster University, Hamilton, Ontario, Canada; Emory University School of Medicine, UNITED STATES

## Abstract

Chronic intestinal inflammation contributes to pathologies such as inflammatory bowel disease (IBD) and colon cancer. While the precise etiology remains controversial, IBD is believed to manifest as a result of various factors. We previously reported that intestinal-specific overexpression of the transcription factor Kaiso results in an intestinal inflammatory response; however, the cause of this inflammation is unknown. To elucidate the underlying mechanism(s) of the Kaiso-mediated intestinal inflammatory phenotype, we evaluated two independent transgenic mouse lines that express varying levels of Kaiso (*Kaiso*^*Tg*^). Histological analyses of *Kaiso*^*Tg*^ mice revealed intestinal damage including thickening of the mucosa, intestinal “lesions” and crypt abscesses, which are reminiscent of IBD pathology. Additionally, higher Kaiso levels induced intestinal neutrophilia as early as 12 weeks, which worsened as the mice aged. Notably, the Kaiso-induced intestinal inflammation correlated with a leaky intestinal barrier and mis-regulation of E-cadherin expression and localization. Interestingly, Kaiso overexpression resulted in reduced proliferation but enhanced migration of intestinal epithelial cells prior to the onset of inflammation. Collectively, these data suggest that Kaiso plays a role in regulating intestinal epithelial cell integrity and function, dysregulation of which contributes to a chronic inflammatory phenotype as mice age.

## Introduction

IBD refers to two intestinal disorders characterized by chronic inflammation: **C**rohn’s **d**isease (CD), which affects both the small and large intestine in discontinuous patches of inflamed lesions, and **u**lcerative **c**olitis (UC), which is restricted to the large intestine and presents as continuous regions of inflamed tissue [1–3]. Currently there is no cure for IBD, and both UC and CD are primarily managed by alleviation of the symptoms [3]. Thus, a better understanding of the underlying molecular and/or genetic factors that contribute to IBD will facilitate the development of a curative treatment.

While the etiology of IBD remains elusive, various mouse models have demonstrated that IBD pathogenesis is multifactorial, with defects in intestinal permeability and intestinal repair mechanisms playing crucial roles. Multi-protein apical adhesion complexes, formed of **t**ight **j**unction**s** (TJs) and **a**dherens **j**unction**s** (AJs), establish the barrier at the apical ends of epithelial cells [4–6]. Compromised intestinal barrier integrity, associated with loss of TJ proteins (e.g. ZO-1, claudins) and AJ proteins (e.g. E-cadherin, p120^ctn^), has been implicated in a leaky intestinal barrier and the subsequent intestinal inflammation [5, 7–13]. Our recent characterization of the novel transgenic mouse overexpressing the transcription factor Kaiso (*Kaiso*^*Tg*^) in the intestines, revealed that at 12-months of age, *Kaiso*^*Tg*^ mice displayed phenotypes consistent with chronic intestinal inflammation; namely, villus blunting, increased **m**yelo**p**er**o**xidase (MPO) levels, and neutrophilia [14].

Kaiso belongs to the family of Pox virus and zinc finger (POZ) **z**inc **f**inger (POZ-ZF) transcription factors, and, like other members of this protein family, Kaiso has roles in vertebrate development and disease [15–25]. Kaiso functions both as a transcription repressor, and as an activator, depending on the tissue microenvironment, but its activity is also regulated by post-translational modifications (e.g. phosphorylation, SUMOylation) [26, 27]. While our study was the first to implicate Kaiso in intestinal inflammation, the incipient stages of this process have not yet been elucidated. We thus sought to further characterize *Kaiso*^*Tg*^ mice to gain insight into the sequence of events leading to the Kaiso-induced inflammatory phenotype.

To this end, we used two independent transgenic mouse lines with differing Kaiso expression levels, (**v**illin **K**aiso line **A**, VKA–moderate Kaiso expression and **v**illin **K**aiso line **E**, VKE–high Kaiso expression) to investigate Kaiso-induced intestinal inflammation. We found that the higher Kaiso-expressing line (VKE) developed inflammation earlier and more extensively than the moderate Kaiso-expressing line (VKA), indicating that high Kaiso expression predisposes the mice to inflammation. To further characterize the Kaiso-induced inflammatory phenotype, pre-symptomatic (12-week old) *Kaiso*^*Tg*^ mice were assessed for changes in the expression and subcellular localization of cell adhesion molecules, as well as for defects in processes that maintain epithelial integrity. Notably, changes in E-cadherin were observed prior to inflammation onset in pre-symptomatic mice. Additionally, pre-symptomatic *Kaiso*^*Tg*^ mice exhibited decreased cell proliferation but increased cell migration before the appearance of intestinal inflammation. Our findings suggest that Kaiso overexpression perturbs the intestinal epithelial integrity via misregulated expression of cell adhesion proteins, which may create an environment that facilitates inflammation.

## Materials & methods

### Ethics statement

All mouse work was conducted according to the guidelines of the McMaster University Animal Research Ethics Board (AREB) and was prospectively approved under the Ethics Approval Number (Animal Utilization Protocol) 14-08-29. *Kaiso*^*Tg*^ mice were generated as previously described [14]. Mice were housed in a 12-hour light/dark cycle and maintained on a standard chow diet. All mice for this study were sacrificed by CO_2_ asphyxiation and cervical dislocation.

### Mouse tissue harvest

Intestines were immediately removed and flushed with cold **p**hosphate-**b**uffered **s**aline (PBS). Small intestines were divided into three equal sections and were either flash frozen or rolled into “Swiss rolls” for fixation in 10% neutral-buffered formalin for 48 hours at room temperature, followed by 70% ethanol dehydration at **r**oom **t**emperature (RT). All analyses were performed on the distal-most regions of the small intestine.

### Immunohistochemistry

A colon disease progression **t**issue **m**icro**a**rray (TMA) was purchased from US Biomax (CO808). This array was comprised of 76 patient cases of which, 4 were Crohn’s disease tissues, 5 were normal intestinal tissues and the remaining 67 represented intestinal adenocarcinomas and metastatic carcinomas. **I**mmuno**h**isto**c**hemistry (IHC) was performed on this TMA and murine intestinal tissues as previously described [14, 24, 28]. The primary antibodies used were as follows: rabbit anti-Ki67 1:150 (Spring Biosciences, Pleasanton, CA); rabbit anti-ZO-1 1:250 (Santa Cruz, Dallas, TX), mouse anti-Claudin2 1:250 (Abcam, Cambridge, MA), mouse anti-p120 catenin (clone 15D2, [29]), mouse anti-β-catenin 1:10,000 (BD Biosciences, Franklin Lakes, NJ), mouse anti-E-cadherin 1:5,000 (BD Biosciences, Franklin Lakes, NJ). Stained tissues were imaged using the Aperio ScanScope slide scanner (Leica Biosystems, Wetzlar Germany), and Ki67-positive cells were scored blind by three independent observers using Image Scope software.

### *In vivo* migration assay

Mice analyzed for **br**omo**d**eoxy**u**ridine (BrdU) label retention were given 50 mg/kg BrdU (Thermo Fisher, Waltham, MA) in PBS by intraperitoneal injection. Mice were sacrificed 24- and 48-hours post-injection, and intestines were harvested as described above. BrdU IHC was performed as stated above using mouse anti-BrdU antibodies at 1:100 dilution (ThermoFisher, Waltham, MA) and the tissues imaged using ImageScope software. To assess cell migration, the distance from the base of the crypt to the forefront-most BrdU-positive cell was measured. Three blind observers quantified the distance migrated from ~ 100 villi (n = 3 mice/genotype).

### Myeloperoxidase (MPO) assay

The MPO activity of 50 mg of flash frozen ileum was analyzed as previously described [14, 24, 28]. Briefly, tissues were homogenized in 0.5% HTAB buffer via sonication at 30 Hz for 4 minutes. Homogenates were centrifuged at 12,000 rpm for 15 min at 4°C. The MPO assay was performed by adding o-dianisidine dihydrochloride solution to the homogenates in triplicate in 96-well plates. Absorbance was measured at 450 nm every 30 sec for 90 sec, in triplicate readings. A total of n = 5–9 mice/genotype per age group were used for each assay.

### Isolation of intestinal *lamina propria* (LP) Cells

LP cells were freshly isolated using collagenase and DNase digestion steps and purified using a Percoll gradient as previously described [30]. Briefly, PBS-flushed intestines were opened longitudinally, cut into ~3 mm pieces and incubated in 1 mM **D**L-di**t**hio**t**hreitol (DTT; Sigma-Aldrich, St. Louis, MO) and 5 mM EDTA in PBS for 10 min in a 37°C shaker to remove mucus. Intestinal sections were vortexed briefly (2–3 sec), passed through a metal sink strainer for collection, and then washed three times in 10% FBS in PBS and 5 mM EDTA for 10 min each in a 37°C shaker to remove the epithelial cell fraction, repeating the collection process as before. After the final wash, the remaining tissue was incubated for 1 hour in a 37°C shaker and digested in 0.5 mg/mL collagenase A (Roche, Indianapolis, IN) with 100 μg/mL DNase I (Roche, Indianapolis, IN) in RPMI media. The samples were filtered through a 70 μm nylon filter in RPMI, releasing LP cells. The cells were collected and centrifuged at 1250 rpm at 4°C for 10 min and resuspended in 6 mL of 40% Percoll (GE Healthcare Life Sciences, Marlborough, MA) in RPMI. Approximately 2 mL of 70% Percoll in RPMI was carefully added to the bottom of the 40% Percoll cell suspension using a long glass micropipette. The samples were centrifuged at 2400 rpm for 30 min at RT, and cells from the 40–70% Percoll interface were transferred to fresh RPMI media. Cells were pelleted at 1300 rpm for 10 min at 4°C, and then resuspended in **f**luorescence-**a**ctivated **c**ell **s**orting (FACS) buffer (0.5% BSA and 0.5M EDTA in PBS) for flow cytometry.

### Flow cytometry

Isolated intestinal LP cells were pre-incubated with anti-CD16/CD32 antibodies (Biolegend, San Diego, CA) for 15 min in the dark on ice to prevent non-specific binding of the fluorochrome-conjugated antibodies. The cells were then incubated with the following fluorochrome-conjugated antibodies for 30 min on ice in the dark: F7/4-FITC (1:200; Abcam, Cambridge, MA), Ly-6G (Gr-1)-PECy5 (1:100; Biolegend, San Diego, CA), CD11b-eFluor605 (1:100; eBioscience, San Diego, CA), F4/80-Pacific Blue (1:100; Biolegend, San Diego, CA), CD45-APC-eFluor780 (1:100; eBioscience), Siglec-F PE (1:200; BD Biosciences, Franklin Lakes, NJ), CD19 PerCPCy5.5 (1:200; eBioscience), NK1.1-PECy7 (1:200; Biolegend), CD3-v500 (1:200; BD Biosciences, Franklin Lakes, NJ), and MHCIIc-ef650 (1:200; eBioscience). Dead cells were excluded by **p**ropidium **i**odide (PI) (Sigma-Aldrich, St. Louis, MO) uptake and gated on singlets. **F**luorescence **m**inus **o**ne (FMO) controls were used for gating. Data were acquired on an LSR II (BD, Franklin Lakes, NJ) and analyzed using FlowJo software (TreeStar Inc., Ashland, OR). A total of n = 7–9 mice/genotype were used for each assay.

### FITC-dextran assay

FITC-dextran (4 kDa; Sigma Aldrich, St. Louis, MO) was diluted to 50 mg/mL in distilled water. Mice were weighed and fasted (food and water) for 7 hours, and then administered 0.6 mg/g bodyweight FITC-dextran solution by intra-gastric gavage. Two hours after intra-gastric gavage, blood was collected via retro-orbital bleeding into whole blood collection tubes. Samples were centrifuged at 12,000 rpm for 3 min at 4°C to separate the plasma, which was analyzed for FITC-dextran levels using a fluorescent spectrophotometer, with excitation at 485 nm and emission at 535 nm (Perkin Elmer Instruments LS Reader Plate Fluorometer, Waltham, MA). Standard curves were generated using known dilutions of FITC-dextran in distilled water and the curves used to calculate the FITC-dextran concentration in all samples. A total of n = 10 mice/genotype were used.

### Isolation of intestinal epithelial cells (IEC)

For IEC isolation, tissues from 6 mice were processed together. Fresh intestinal tissues were harvested from sacrificed mice as stated above and flushed with ice-cold saline solution (5 M NaCl, 1 M imidazole, pH 7.2, 10% sodium azide). Small intestinal segments were opened longitudinally, cut into 2 cm sections and stirred vigorously in ice-cold sucrose buffer (200 mM sucrose, 20 mM KH_2_PO_4_ monobasic, 80 mM Na_2_HPO_4_ dibasic, 0.5 M EDTA, 10% sodium azide) at 4°C for ≥ 2 hours. The sucrose buffer with isolated epithelial cells was poured through a metal strainer to remove large tissue sections and pelleted at 400 g 4°C for ≥ 10 min. IEC pellets were then washed 3 times with cold sucrose buffer, once with cold PBS, and then stored at -80°C until further processing. qPCR and western blot experiments utilizing isolated IECs were performed in experimental triplicate.

### Immunoblot

IEC pellets were resuspended, lysed and denatured using Laemmli sample buffer (2% SDS, 5% β-mercaptoethanol, 62.5 mM Tris, pH 6.8) and boiled for 5 min. Lysates were spun at 14,000 rpm for 30 min at 4°C, and protein concentration was determined by DC Protein Assay, as per manufacturer’s instructions (BioRad, Hercules, CA). Equal amounts of protein were subjected to SDS-PAGE and proteins were transferred to a nitrocellulose membrane, blocked in 3% milk/TBS and incubated at 4°C overnight with the following antibodies: rabbit anti-Kaiso 1:1,000 (gift from Dr. Albert Reynolds), mouse anti-β-catenin 1:40,000 (BD Biosciences, Franklin Lakes, NJ), mouse anti-E-cadherin 1:10,000 (BD Biosciences, Franklin Lakes, NJ) mouse anti-p120^ctn^ 1:5000 (clone 15D2, [29]), rabbit anti-cyclin D1 (US Biologicals, Salem, MA), rabbit anti-cleaved-Caspase 3 1:1000 (Cell Signaling Technology, Beverly, MA), mouse anti-β-actin 1:50,000 (Sigma-Aldrich, St. Louis, MO). Membranes were washed 5X for 5 min with TBS and incubated at RT for 2 hours with the appropriate HRP-conjugated secondary antibody. Membranes were washed again as described above to remove excess secondary antibody and developed using Clarity Western ECL Substrate (BioRad, Hercules, CA). Images were taken using BioRad ChemiDoc MP, and processed using Image Lab software (BioRad, Hercules, CA).

### Quantitative RT-PCR

Total RNA was isolated from IEC pellets using the NucleoSpin RNA isolation kit according to manufacturer’s instructions (Macherey-Nagel, Bethlehem, PA). mRNA was reverse-transcribed into cDNA using SensiFAST cDNA Synthesis Kit according to manufacturer’s protocol (Bioline, Taunton, MA), and qRT-PCR was performed using the SensiFAST SYBR Hi-ROX Kit (Bioline, Taunton, MA). The relative expression of macrophage inflammatory protein 2 (MIP-2) was analyzed using the standard curve method with **h**ydroxy**m**ethyl**b**ilane **s**ynthase (HMBS) as the endogenous control. The primer sequences used were as follows: *MIP-2 Fwd*: *5’ CAC TCT CAA GGG CGG TCA AA 3’; MIP-2 Rev*: *5’ GGT TCT TCC GTT GAG GGA CA 3’; mHMBS Fwd*: *5’ GAT GGG CAA CTG TAC CTG ACT G 3’; mHMBS Rev*: *5’ CTG GGC TCC TCT TGG AAT G 3’*.

### Statistical analyses

Where indicated, Student’s t-test, one-way ANOVA or two-way ANOVA analysis was used to determine statistical significance (p < 0.05) using GraphPad Prism 6.0 software (La Jolla, CA).

## Results

### Kaiso expression is increased in human inflammatory bowel disease

**Fig 1:**
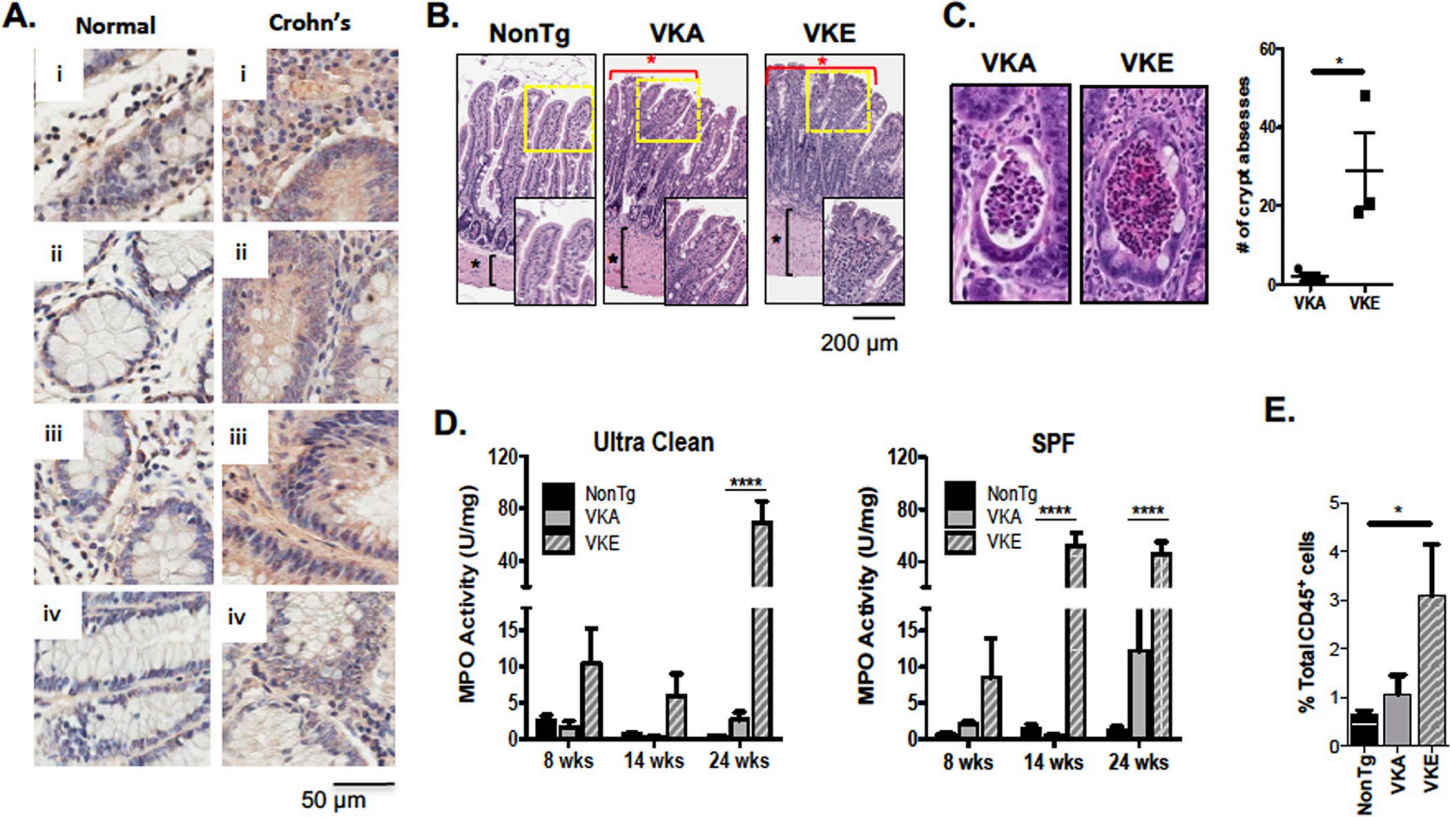
*Kaiso*^*Tg*^ mice display hallmarks of chronic inflammation. **(A)** Inflammation in Crohn’s disease (CD) patients correlates with higher Kaiso expression levels. **(B)** *Kaiso*^*Tg*^ mice display discontinuous lesions through the small intestine (red asterisks), blunted villi and thickening of the *muscularis externa* (black asterisks) compared to the NonTg siblings at 8-months of age. Insets are enlarged images of the areas outlined in yellow. **(C)** Line A (VKA) shows fewer crypt abscesses at 8 months of age compared to Line E (VKE) (n = 3 mice/phenotype). Statistical significance was determined by student’s *t*-test, and error bars are SEM *p <0.05. **(D)** Early age MPO analysis on age matched VKA and VKE mice show an increase in inflammation in VKE mice compared to VKA and NonTg mice at 14 weeks in SPF (n = 5–7 mice/genotype per age). Statistical significance was determined by two-way ANOVA, and error bars are SEM, ****p <0.0001. **(E)** At 14 weeks, VKE mice show a significant increase in neutrophils compared to age-matched NonTg mice and VKA mice. (n = 7–9 mice/genotype). Statistical significance was determined by one-way ANOVA, and error bars are SEM, *p <0.05.

Our finding that intestinal-specific Kaiso overexpression results in spontaneous chronic inflammation in 12-month old mice [14] prompted us to examine Kaiso expression levels in human Crohn’s disease (CD) tissues. Using IHC, we examined Kaiso expression and subcellular localization in human normal and Crohn’s disease intestinal tissues and observed cytoplasmic and nuclear Kaiso localization in both normal and CD tissues. Importantly, we detected enhanced Kaiso staining in all four CD intestinal tissues (US Biomax) compared to normal tissues (Fig 1A), supporting the notion that Kaiso may be a relevant participant in the development of chronic intestinal inflammation.

### Kaiso-induced intestinal inflammation leads to long-term tissue damage

To further characterize the Kaiso inflammatory phenotype, two **v**illin-***K****aiso*^*Tg*^ (VK) mouse lines were examined: the moderate Kaiso-expressing Line A (VKA) and the high Kaiso-expressing Line E (VKE; S1 Fig) [14]. Both VKA and VKE mice displayed a thickened *muscularis externa* at ~8 months of age (Fig 1B) and exhibited discontinuous “lesions” throughout the length of the small intestine separated by healthy villi. These lesions were comprised of blunted and fused villi, and elevated immune cell infiltration into the underlying LP (Fig 1B, S2 Fig). While evidence of Kaiso-induced inflammation was widespread by 8-months of age, we focused our analyses on the ileum, as this region of the small intestine developed signs of inflammation earlier than the duodenum and jejunum and hence had the most severe phenotype.

Among the discerning phenotypes associated with IBD are crypt abscesses ─ aggregations of polymorphonuclear leukocytes, especially neutrophils, in the crypt lumen [31]. By ~8 months of age, both *Kaiso*^*Tg*^ lines (n = 3/genotype) formed crypt abscesses in the ileum, although VKE mice developed significantly more crypt abscesses compared to VKA mice (Fig 1C). No crypt abscesses were detected in the intestines of age-matched non-transgenic (NonTg) littermates. Collectively, these data highlight that intestinal-specific Kaiso overexpression facilitates intestinal inflammation, consistent with our previous findings [14, 24].

### High Kaiso expression induces neutrophil infiltration and activation

Since *Kaiso*^*Tg*^ mice display chronic inflammation and tissue damage associated with increased immune cell infiltration, we assessed whether microbial load from the environment influences the development of the intestinal inflammation observed in *Kaiso*^*Tg*^ mice. MPO activity, a surrogate marker of activated neutrophils [32], was measured in 8- and 14-week old mice housed in two conditions: an ultraclean room (low microbial load), or the common **s**pecific **p**athogen-**f**ree (SPF) room (Fig 1D). While there was no difference in the neutrophil activity of 8- or 14 week old VKA mice in either housing condition, VKE mice presented with elevated MPO activity at 8 weeks under both housing conditions, which significantly increased at 14 weeks of age under SPF conditions (Fig 1E). The early manifestation of MPO activity in VKE mice suggests that high Kaiso expression enhances susceptibility to inflammation, which is influenced by the environmental microbial load.

We next evaluated the presence of neutrophils (CD45^+^) in the LP of the small intestine at 14 weeks when VKE, but not VKA mice, have ongoing inflammation. Using flow cytometry analysis, we determined that 14-week old VKE mice displayed significantly increased neutrophil infiltration into the LP, in contrast to VKA, which only showed a slight increase (Fig 1E). However, at 8 months of age VKA mice developed a significant increase in neutrophils with no significant change in the numbers of other immune cell types (S3 Fig). Together, these data suggest that elevated Kaiso expression induces an early onset of intestinal inflammation that is neutrophil-specific and environment-dependent.

**Fig 2:**
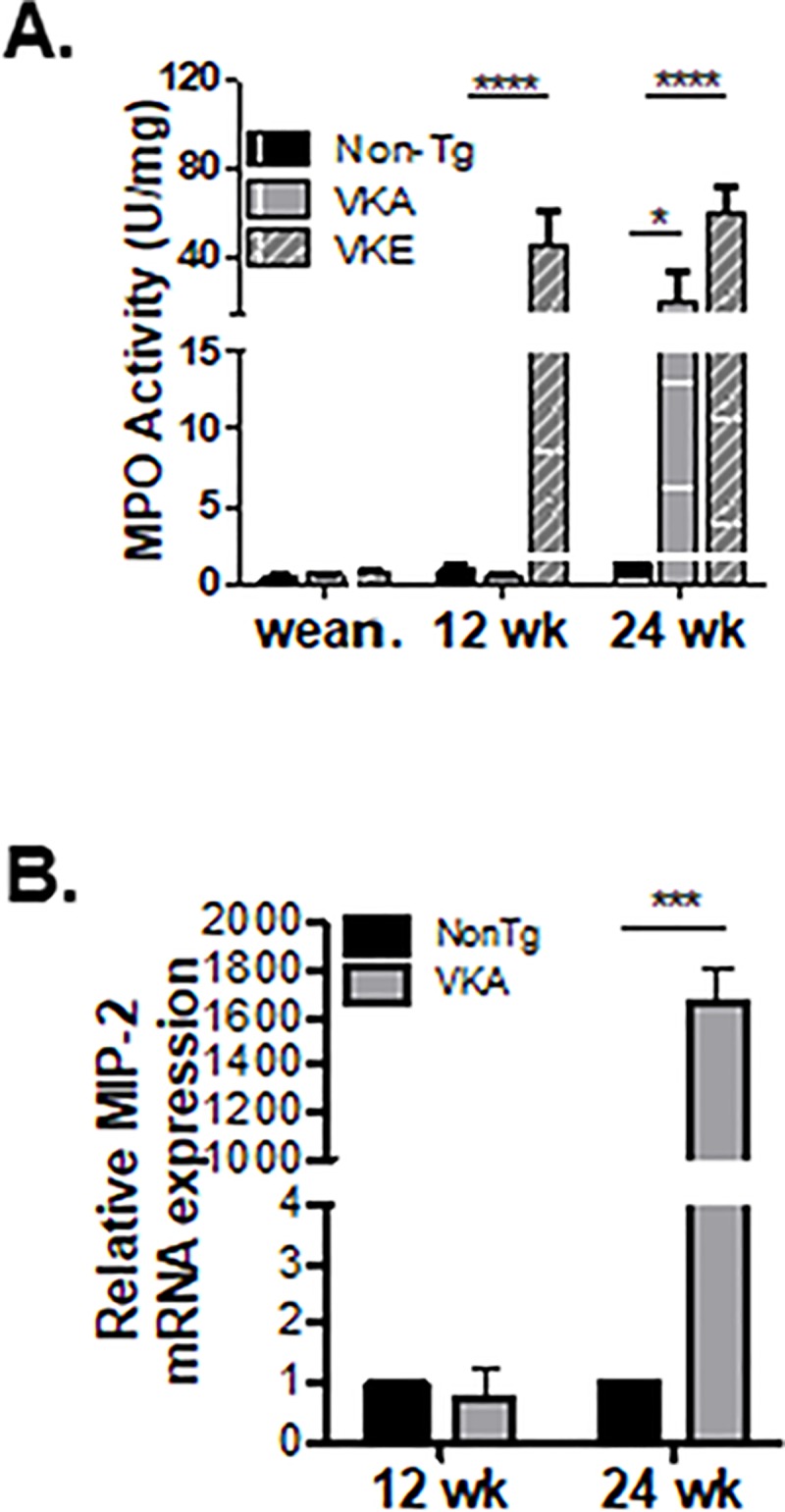
*Kaiso*^*Tg*^ mice develop inflammation with age. **(A)** *Kaiso*^*Tg*^ mice do not show inflammation upon weaning (~3 wk.). However, Line E (VKE) mice develop inflammation faster than Line A (VKA) mice (12- vs. 24-wks of age; n = 5–9 mice/genotype/time point). Statistical significance was determined by two-way ANOVA, and error bars are SEM; * p<0.05; ****p <0.0001. **(B)** mRNA expression levels of the neutrophil attracting cytokine, MIP-2, are increased after, but not before, inflammation in VKA mice compared to NonTg mice, at 24- and 12-weeks of age, respectively. qPCR data are of pooled biological replicates from IECs isolated from the entire small intestine of 6 mice/genotype for each time point, run in experimental and technical triplicate. Statistical significance was determined by student’s *t*-test, and error bars are SEM; ***p <0.001.

To determine the timeline of the neutrophil-specific inflammation in both VKA and VKE mice, MPO activity was assayed at various time points: 3- (weaning), 12- and 24-weeks of age (Fig 2A). Neither VKA nor VKE mice exhibited inflammation (as assessed by MPO-activity) upon weaning, although VKE mice displayed significant MPO activity by 12 weeks of age. While neutrophil activity was significantly elevated in both VKA and VKE mice by 24 weeks, VKE mice exhibited significantly more MPO activity than their VKA counterparts (Fig 2A).

The potent neutrophil chemokine, MIP-2, is secreted by various cell types including IECs [33, 34]. As a first step in evaluating the underlying mechanism(s) of Kaiso-induced neutrophilia, we examined MIP-2 mRNA expression levels in age-matched NonTg and *Kaiso*^*Tg*^ IECs. To determine if there was a change in MIP-2 levels prior to or after neutrophilia, comparisons were focused on pre-symptomatic (12-week old) and diseased (24-week old) VKA mice (Fig 2A). VKE mice were not examined since they exhibit enhanced MPO activity as early as 8-weeks, and significantly elevated MPO levels by 12-weeks of age (Figs 1E & 2A). mRNA from IECs was isolated from age-matched VKA and NonTg littermates (n = 6/genotype), and qRT-PCR for *MIP-2* was performed. Consistent with MPO activity data (Fig 2A), diseased VKA mice have increased *MIP-2* expression at 24 weeks, but not at 12-weeks, compared to their age-matched NonTg counterparts (Fig 2B). Taken together, these data indicate that Kaiso overexpression enhances neutrophil recruitment and degranulation within the intestines and suggest that changes in *MIP-2* expression, while not a direct cause of neutrophilia, aggravate the neutrophilia phenotype in the inflamed microenvironment.

### *Kaiso*^*Tg*^ mice exhibit hallmarks of a defective intestinal epithelial barrier upon inflammation

**Fig 3:**
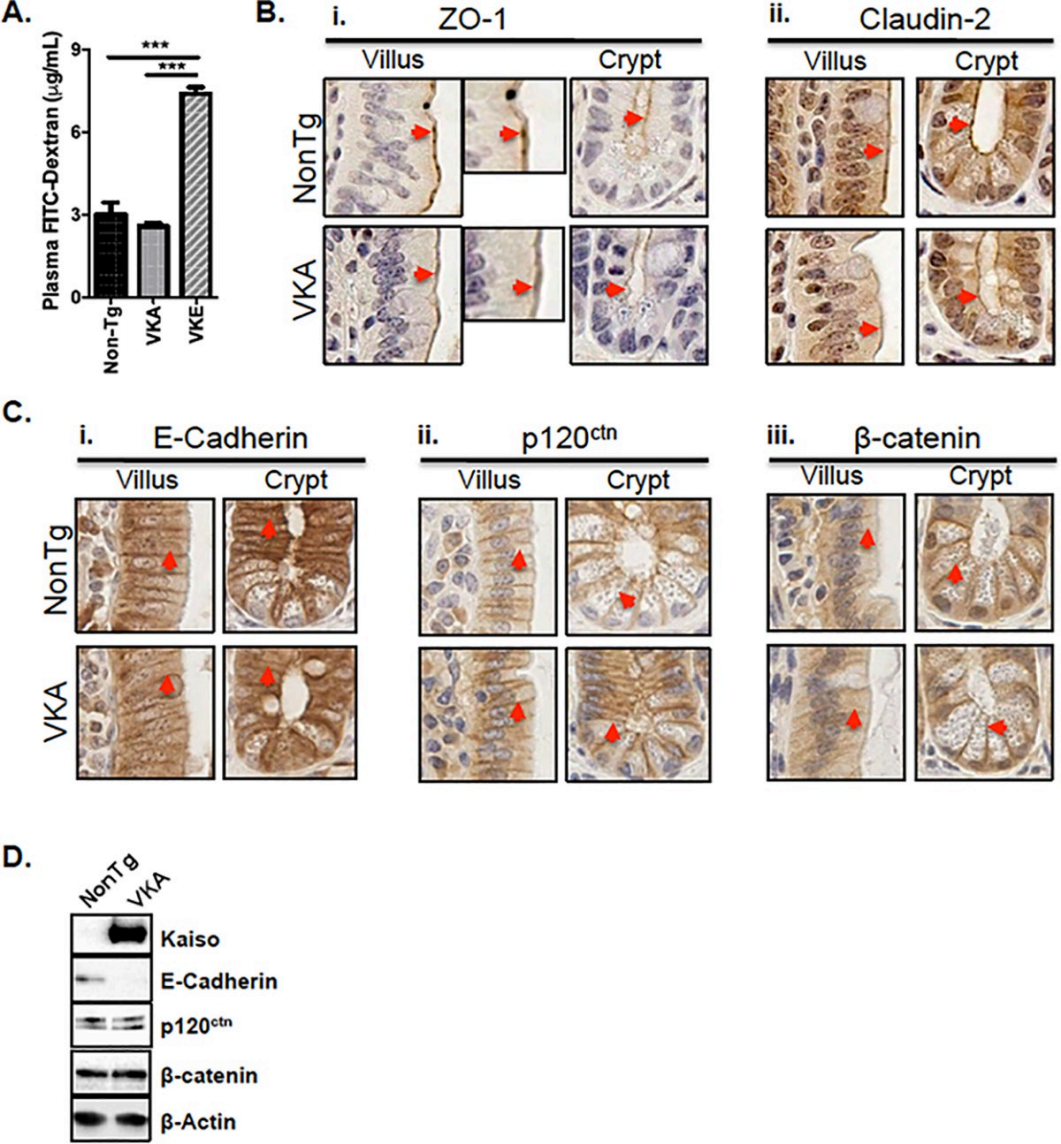
Pre-symptomatic VKA mice exhibit reduced E-cadherin expression prior to inflammation onset. **(A)** Inflamed intestinal tissues of 14-week old VKE mice exhibit significantly increased permeability to 4 kDa FITC-dextran, compared to the non-inflamed tissues of age-matched VKA and NonTg mice (n = 10 mice/genotype). Statistical significance was determined by one-way ANOVA, ****p<0.0001, error bars represent SEM. **(B)** IHC of ZO-1 and Claudin-2 show similar localization to the apical membrane (red arrowheads) in both 12-week old NonTg and VKA mice. **(C)** IHC of E-Cadherin, p120^ctn^ and β-catenin reveals localization along the lateral membrane (red arrowheads) in both 12-week old NonTg and VKA mice. **(C-i)** 12-week old VKA mice display a modest reduction in laterally-localized E-cadherin in both villi and crypts, compared to NonTg mice prior to inflammation onset, however Kaiso overexpression did not alter the expression or subcellular localization of either p120^ctn^ or β-catenin **(C-ii, iii)**. **(D)** Immunoblot analysis of adherens junction proteins from isolated IECs reveals decreased levels of E-cadherin, but not p120^ctn^ or β-catenin, in 12-week old VKA mice compared to NonTg siblings.

Intestinal inflammation is often associated with a compromised intestinal epithelial barrier and an augmented intestinal permeability [35]. In light of our findings that Kaiso overexpression results in intestinal inflammation, we next interrogated the integrity of the intestinal epithelial barrier of *Kaiso*^*Tg*^ mice using a FITC-dextran assay. At 14-weeks of age, a significant increase in plasma FITC-dextran was detected in SFP-housed VKE mice, suggesting that the enhanced gut permeability may be due to a defective intestinal epithelial barrier (Fig 3A). Notably, pre-symptomatic age-matched VKA mice did not exhibit any permeability changes, implying that the severe inflammation and intestinal barrier defect observed in VKE mice are due to higher Kaiso expression.

Loss of the Kaiso binding partner and E-cadherin co-factor, p120^ctn^, results in spontaneous inflammation in mice [10, 11]. Since we and others recently showed that Kaiso-depletion causes de-repression of epithelial proteins such as E-cadherin and ZO-1 in breast and prostate cancer cell lines [15, 36], we postulated that Kaiso overexpression may modulate adhesion protein expression in the intestinal epithelium. To test this hypothesis, the expression and subcellular localization of several cell adhesion proteins were compared in pre-symptomatic (12-week old) VKA mice relative to their NonTg counterparts. Using IHC, we first examined the expression and localization of tight junction proteins, ZO-1 and Claudin-2. In 12-week old NonTg mice, ZO-1 localized to the apical membrane in IECs of both the villus and crypt and is visible as distinct puncta between neighboring cells in the villus (Fig 3B-i). Pre-symptomatic 12-week old VKA mice likewise exhibited apically localized ZO-1 in both the villus and crypt, but distinct ZO-1 puncta between neighboring villus cells were not observed (Fig 3B-i). Apically localized Claudin-2 was detected primarily in the crypts of both NonTg and VKA mice at 12-weeks of age, but Claudin-2 expression levels appeared relatively unchanged (Fig 3B-ii).

IHC was also performed to assess the subcellular localization of E-cadherin, p120^ctn^ and β-catenin. All three adherens junction proteins localized to the lateral membrane in both genotypes prior to inflammation at 12-weeks old (Fig 3C). While no detectable change in p120^ctn^ or β-catenin localization was observed between VKA and NonTg in the villi or crypts (Fig 3C-ii & 3C-iii), 12-week old VKA mice displayed reduced E-cadherin at the lateral cell membrane in both cellular compartments compared to age-matched NonTg littermates (Fig 3C-i). Immunoblot analysis on adherens junction proteins also revealed a reduction in E-cadherin expression in isolated IECs from pre-symptomatic VKA mice (Fig 3D). Since only E-cadherin localization and expression was perturbed prior to measurable inflammation (i.e. in 12-week old mice), these data suggest that reduced E-cadherin at the lateral membrane may perturb the epithelial barrier and contribute to subsequent intestinal inflammation as mice age.

### Pre-symptomatic *Kaiso*^*Tg*^ mice exhibit altered intestinal renewal mechanisms

**Fig 4:**
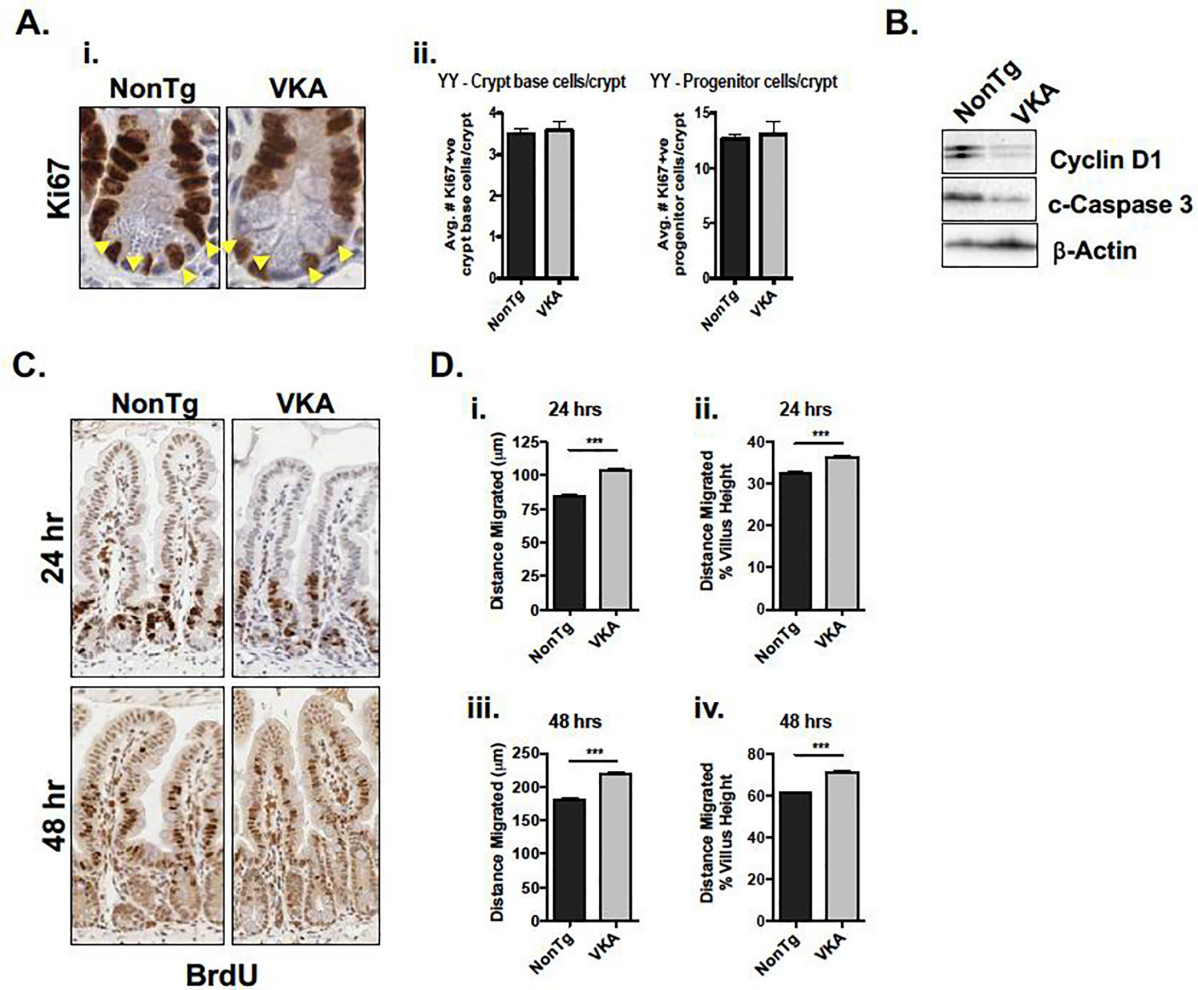
Intestinal epithelial cell repair mechanisms are altered prior to inflammation onset in 12-week old VKA mice. **(A-i)** VKA mice do not demonstrate a change in number of Ki67-positive cells. Yellow arrowheads denote crypt base cells. **(A-ii)** Ki67-positive cells from 100 open crypts (n = 5 mice/genotype) were quantified. Crypt base cells were scored by counting the number of Ki67-positive cells in the bottom third of the crypt. The remaining cells were scored as progenitor cells. **(B)** Proliferation marker Cyclin D1 and apoptotic marker cleaved (c-) Caspase 3 are both decreased in VKA mice. **(C, D)** VKA mice show increased migration of epithelial cells up the crypt-villus axis relative to their NonTg counterparts, as demonstrated by IHC **(C)**, and quantified by measuring the distance of the furthest-migrated BrdU-positive cell (~ 100 villi from 3 mice/genotype) **(D). (D-i, -iii)** Distance migrated was quantified by measuring the distance from the base of the crypt, to the furthest positively BrdU-labelled cell. **(D-ii, iv)** To correct for possible differences in villus height, the average distance migrated was normalized to the average villus height. Representative plots of at least three blind measurements are shown. Statistical significance was determined by unpaired student’s *t*-test. ***p<0.001, error bars represent SEM.

Intestinal renewal is important for healing damage to the epithelium caused by inflammation. This process is achieved by the delicate balance between generation of new cells by proliferation, their migration up the crypt-villus axis, followed by their subsequent apoptosis once cells reach the villus tip [35]. We therefore examined cell proliferation in 12-week old VKA and NonTg mice as a first step in determining whether the regenerative capacity of the intestinal epithelium was perturbed in VKA mice. To this end, intestines were stained for the proliferation marker, Ki67, by IHC. Positively-labelled crypt base cells (localized at the bottom ¼ of the crypt), were quantified separately from presumed progenitor cells. Kaiso overexpression did not result in a change in either population of cells (Fig 4A). We next compared the protein expression levels of cell proliferation and apoptosis markers from isolated IECs of 12-week old NonTg and VKE or VKA mice. Consistent with previous findings [14, 19, 37, 38], 12-week old VKA mice displayed diminished Cyclin D1 protein expression relative to their NonTg siblings (Fig 4B). However, since expression levels and subcellular localization of the Wnt effector β-catenin were unchanged in 12-week old VKA mice (Fig 3C and 3D), the change in Cyclin D1 expression is unlikely due to changes in canonical Wnt signalling. Examination of the apoptotic marker, cleaved Caspase 3 (c-Caspase 3) revealed reduced expression in VKA mice prior to inflammation onset (Fig 4B). Although the Ki67 index was unchanged by Kaiso-overexpression, the finding that pre-symptomatic VKA mice exhibited altered Cyclin D1 and c-Caspase 3 expression indicate abnormal intestinal renewal processes in 12-week old VKA mice, independently of Wnt/β-catenin signalling.

Given our findings that E-cadherin expression and lateral localization are diminished in pre-symptomatic VKA mice, and since E-cadherin is a Kaiso target gene [15, 17] and is an important regulator of collective cell migration during intestinal restitution [39], we next investigated whether IEC migration was altered in VKA mice. To ascertain the migratory capacity of the epithelium of 12-week old VKA mice and to determine whether IEC migration was affected prior to inflammation onset, pre-symptomatic 12-week old VKA and age-matched NonTg mice were injected intraperitoneally with BrdU and euthanized 24- and 48-hours post-injection. In this setting, BrdU is incorporated by actively proliferating stem cells, and retained in non-proliferating differentiated IECs [40]. Thus, the location of BrdU-retaining cells along the crypt-villus axis has been used as a measure of cell migration [40]. The distance travelled by BrdU-positive cells was quantified from ~100 villi from 3 mice/genotype (Fig 4C). By 24-hours post-injection, we observed a significant increase in the migration of BrdU-retaining VKA intestinal cells compared to NonTg intestinal cells (104.0 ± 1.66 μm *versus* 84.89 ± 1.14 μm, p<0.001, Fig 4D). A similar trend was observed at the 48-hour time-point, as BrdU-retaining VKA intestinal cells had migrated significantly further compared to those of their NonTg siblings (218.9 ± 2.97 μm *versus* 180.3 ± 2.60 μm, Fig 4D). Collectively, these data demonstrate that the replenishing capacity of the *Kaiso*^*Tg*^ epithelium is perturbed prior to measurable signs of inflammation. Specifically, Kaiso overexpression delays overall proliferation and apoptosis, but enhances IEC migration, which together may ultimately affect the integrity and function of the intestinal epithelium.

## Discussion

We previously demonstrated that intestinal-specific overexpression of the transcription factor Kaiso facilitates a chronic inflammatory phenotype in mice [14, 24], suggesting that Kaiso may be important in IBD pathogenesis. The observation that Kaiso expression is elevated in intestinal tissues of patients with CD (Fig 1) supported our hypothesis that Kaiso may potentiate inflammation and play a role in IBD disease progression. This necessitated a more detailed characterization of the mechanism(s) contributing to the Kaiso-induced inflammatory phenotype.

A number of studies have attributed intestinal inflammation to a loss of apical junctional proteins, such as E-cadherin [12], p120^ctn^ [10, 11] and ZO-1 [5]. We previously reported enhanced nuclear p120^ctn^ localization in 1 year-old VKA mice [14], which was consistent with other studies demonstrating intestinal inflammation in p120^ctn^ null tissues [10, 11]. However, the fact that 12-week old mice did not exhibit a change in p120^ctn^ localization (Fig 3) suggests that altered localization of p120^ctn^ develops over time. Additionally, our observation of diminished E-cadherin levels is in agreement with multiple reports demonstrating Kaiso-mediated transcriptional repression of E-cadherin expression [15, 36, 41]. Given the essential role of E-cadherin in maintaining intestinal epithelial barrier integrity [12], our findings suggest that loss of E-cadherin in VKA mice contributes to a weaker epithelial barrier than their NonTg littermates. Importantly, while E-cadherin is reduced in pre-symptomatic VKA, some barrier function is maintained at this time point, since permeability to 4 kDa FITC-dextran was unchanged in these mice. Hence, a fully defective barrier appears to depend on both age and levels of Kaiso expression, as highly-overexpressing VKE mice exhibit more severe barrier defects at an earlier age than moderately over-expressing VKA mice [14]. Our group and others have shown that Kaiso regulates E-cadherin, both by binding to the *CDH1* locus, and by regulating TGF-β signaling [15, 36, 41, 42]. Therefore, one possible mechanism by which Kaiso might inhibit E-cadherin in pre-symptomatic VKA mice is by repression of the *CDH1* locus and/or modulation of the TGF-β pathway.

Continued neutrophil recruitment and migration across the epithelial barrier has been shown to contribute to the pathogenesis of colitis [7, 32]. Neutrophil-mediated lesions, such as crypt abscesses, have also been postulated to contribute to intestinal leakiness as neutrophil transmigration has been shown to create gaps in the epithelial lining [7, 43]. The observed dose-dependent increase in MPO activity, enhanced transcript levels of the neutrophil chemoattractant, MIP-2 (Fig 2), and the formation of crypt abscesses (Fig 1) in diseased *Kaiso*^*Tg*^ mice, support the notion that neutrophil recruitment contributes to Kaiso-induced inflammation.

The finding that pre-symptomatic 12-week old VKA mice exhibited altered IEC migration and proliferation (Fig 4) suggests that abnormal intestinal renewal mechanisms may contribute to the pathogenesis of chronic inflammation in *Kaiso*^*Tg*^ mice. While some studies suggest that proliferation in the crypt propels IEC migration [44], others indicate that proliferation and migration occur independently, and that active collective cell migration drives the movement of cells up the crypt-villus axis [45, 46]. Our results are consistent with the latter hypothesis, which supports uncoupled intestinal proliferation and migration. The accelerated IEC migration in *Kaiso*^*Tg*^ mice may be explained by the findings that Kaiso promotes expression of TGFβR-I and -II [15], which are well-known for their pro-migratory roles [47], and/or the observed reduction in E-cadherin at the lateral membrane, which might be due by Kaiso-mediated transcriptional repression of E-cadherin [15, 36, 41, 42] (Fig 3). Kaiso is also implicated in the regulation of other factors and pathways necessary for intestinal cell proliferation and turnover including, Cyclin D1 [19, 20, 37, 38], p53 [16, 48, 49], and the Notch signalling pathway [28]. As such, we cannot exclude the possible contributions each of these may have on the observed phenotypes in *Kaiso*^*Tg*^ mice.

## Conclusion

**Fig 5:**
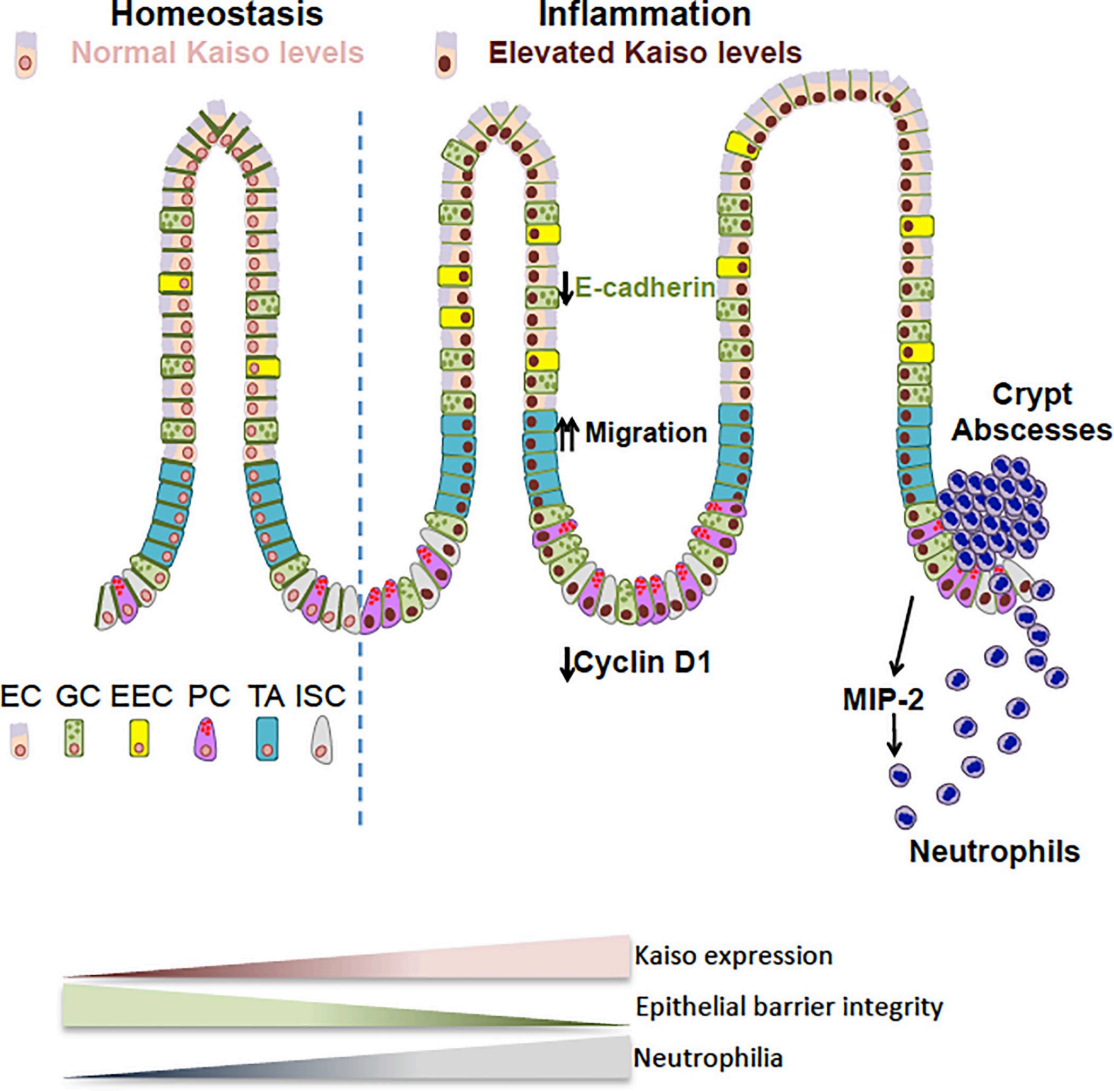
Working model of kaiso-mediated intestinal inflammation. Pre-symptomatic *Kaiso*^*Tg*^ mice exhibit attenuated E-cadherin expression, which is accompanied by accelerated IEC migration along the crypt-villus axis. Together with diminished Cyclin D1 expression, this suggests that IECs migrate faster than they are turned over. Poor intestinal epithelial integrity renders *Kaiso*^*Tg*^ mice susceptible to microbial infection, which triggers a neutrophil-specific response. Pathogenic neutrophil recruitment results in continued intestinal tissue damage (e.g. blunted villi, crypt abscesses etc.), and thus exacerbates inflammation. EC–enterocyte, GC–goblet cell, EEC–enteroendocrine cell, PC–Paneth cell, TA–transit amplifying cell, ISC–intestinal stem cell.

We report that Kaiso-induced intestinal inflammation involves perturbation of intestinal epithelial cell integrity, which is subsequently followed by a chronic inflammatory phenotype. We propose a working model of Kaiso-mediated inflammation, whereby reduced membrane localization of E-cadherin and abnormal epithelial renewal processes render the epithelium susceptible to inflammation and subsequent neutrophil recruitment. Together these perturbations ultimately contribute to increasingly severe chronic intestinal inflammation as *Kaiso*^*Tg*^ mice age. Since the onset of neutrophil activity in *Kaiso*^*Tg*^ mice varies depending on housing conditions, environmental factors (e.g. microbial load) may also influence the development of chronic inflammation in these mice, which is an avenue of future research (Fig 5). Nonetheless, the occurrence of spontaneous chronic inflammation observed in *Kaiso*^*Tg*^ mice makes this mouse model a novel *in vivo* tool for IBD research.

## Supporting information

S1 Fig Kaiso expression levels in two independent Kaiso-overexpressing mouse lines. Both villin Kaiso line A (VKA) and line E (VKE) mice express more Kaiso than non-transgenic mice, however VKE mice express substantially more Kaiso than their VKA counterparts.
(TIF)

S2 Fig Kaiso overexpression causes widespread inflammation in all parts of the small intestine by 8-months of age. Evidence of inflammation, including elevated immune cell infiltration into the *lamina propria*, and blunted and fused villi, was observed in the duodenum and jejunum of *Kaiso*^*Tg*^ mice. Images are 10X magnification, and insets are enlarged images of the selected regions.
(TIF)

S3 Fig Evaluation of leukocytes in 8-month old VKA mice. **(A)** Leukocytes evaluated in VKA at 8 months of age display no differences in macrophages (CD11b^+^ F4/80^+^), eosinophils (Siglec-F^+^), dendritic (MHCII^+^ CD11c^+^), NK (NK1.1^+^), T (CD3^+^) or B (CD19^+^) cells, while **(B)** VKA mice exhibit neutrophilia (n = 8 mice/genotype). Statistical significance was determined by student *t*-test, and error bars are SEM ***p <0.05.
(TIFF)
