# Supplementary figures and images for: Kaiso-induced intestinal inflammation is preceded by diminished E-cadherin expression and intestinal integrity
Source: PLoS One. 2019 Jun 14;14(6):e0217220. doi: 10.1371/journal.pone.0217220 (PMC6568390; doi:10.1371/journal.pone.0217220)

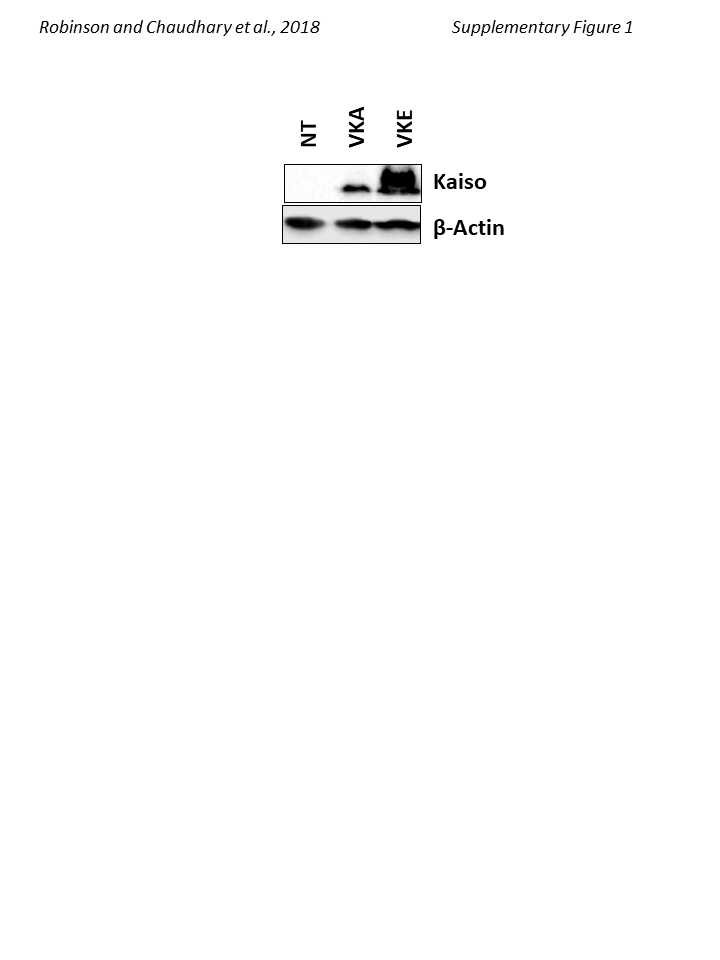

Supplement: S1 Fig — Both villin Kaiso line A (VKA) and line E (VKE) mice express more Kaiso than non-transgenic mice, however VKE mice express substantially more Kaiso than their VKA counterparts. (TIF) [file pone.0217220.s001.tif]

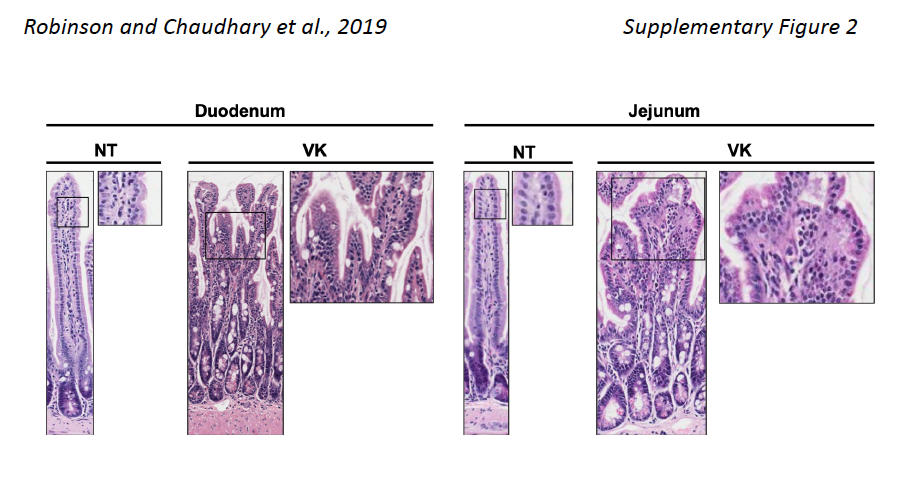

Supplement: S2 Fig — Evidence of inflammation, including elevated immune cell infiltration into the lamina propria, and blunted and fused villi, was observed in the duodenum and jejunum of KaisoTg mice. Images are 10X magnification, and insets are enlarged images of the selected regions. (TIF) [file pone.0217220.s002.tif]

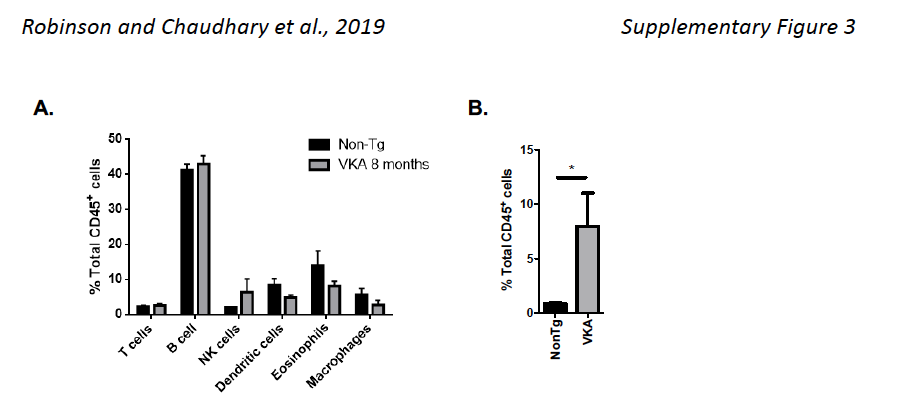

Supplement: S3 Fig — (A) Leukocytes evaluated in VKA at 8 months of age display no differences in macrophages (CD11b+ F4/80+), eosinophils (Siglec-F+), dendritic (MHCII+ CD11c+), NK (NK1.1+), T (CD3+) or B (CD19+) cells, while (B) VKA mice exhibit neutrophilia (n = 8 mice/genotype). Statistical significance was determined by student t-test, and error bars are SEM ***p <0.05. (TIFF) [file pone.0217220.s003.tiff]
